# Splice effect of a synonymous variant in *AP4B1*: multiomics approach establishes the diagnosis in two sisters with spastic paraplegia

**DOI:** 10.1155/humu/8645235

**Published:** 2026-08-12

**Authors:** Susann Badmann, Alice Saparov, Philip Harrer, Juliane Beuschlein, Cornelia Daumer-Haas, Elisabeth Graf, Christina Ludwig, Julia Mergner, Theresa Brunet, Maureen Jacob, Holger Prokisch, Juliane Winkelmann, Thomas Meitinger, Michael Zech, Matias Wagner

**Affiliations:** ^1^ Institute of Human Genetics, School of Medicine and Health, Technical University of Munich, Munich, Germany, tum.de; ^2^ Bavarian Genomes Network for Rare Disorders, Bavaria, Germany; ^3^ Institute of Neurogenomics, Helmholtz Munich, Neuherberg, Germany; ^4^ Helmholtz Association-Munich School for Data Science (MUDS), Munich, Germany; ^5^ Eurofins Humangenetik und Praenatal-Medizin MVZ GmbH, Munich, Germany; ^6^ Institute of Medical Genetics and Human Genetics, Charité-Universitaetsmedizin Berlin, Corporate member of Freie Universitaet Berlin and Humboldt-Universitaet zu Berlin, Berlin, Germany, charite.de; ^7^ Bavarian Center for Biomolecular Mass Spectrometry (BayBioMS), Technical University of Munich, Freising, Germany, tum.de; ^8^ Bavarian Center for Biomolecular Mass Spectrometry at the University hospital rechts der Isar, Technical University of Munich, Munich, Germany, tum.de; ^9^ Division of Pediatric Neurology, Department of Pediatrics, Developmental Medicine and Social Pediatrics, Dr. von Hauner Children′s Hospital, Ludwig Maximilian University Munich, Munich, Germany; ^10^ Munich Cluster for Systems Neurology (SyNergy), Munich, Germany, synergy-munich.de; ^11^ Institute for Advanced Study, Technical University of Munich, Garching, Germany, tum.de

**Keywords:** *Ap4b1*, hsp, proteomics, rna sequencing, splice effect, synonymous variant

## Abstract

Patients with suspected monogenic disorders often remain undiagnosed after exome sequencing. We report a family with two sisters affected by a complex spastic paraplegia. Initial exome sequencing had identified monoallelic pathogenic nonsense variants in *AP4S1* and *AP4B1*, subunits of the adaptor protein complex 4 (AP‐4), suggesting digenic inheritance. As digenic inheritance has not been established for AP‐4–associated disorders, we applied a multiomics approach including genome sequencing, RNA sequencing and proteomics to clarify the genetic cause. By RNA sequencing a predicted synonymous variant (NM_006594.5:c.969G > A), compound heterozygous to the nonsense variant in *AP4B1* and previously considered as benign, was re‐prioritized as aberrant splicing was demonstrated. Proteomics showed reduced abundance of AP‐4 components AP4B1 and AP4M1 and an upregulation of the cargo protein ATG9A, confirming AP‐4 deficiency. Although the *AP4S1* variant resulted in nonsense‐mediated decay, the identification of biallelic causative variants in *AP4B1* established the diagnosis of monogenic “Spastic paraplegia 47, autosomal recessive” while the initial hypothesis of digenic inheritance was refuted. This study illustrates the value of multiomics approaches in the diagnostic workflow of rare diseases and the potential for pathogenicity of synonymous variants.

## 1. Introduction

Individuals with suspected monogenic disorders often remain undiagnosed after whole exome sequencing (WES) as the diagnostic yield remains below 50% [1, 2]. Hereditary spastic paraplegia (HSP) comprises genetically heterogeneous neurodegenerative disorders involving degeneration of corticospinal tract axons. Clinically, HSP is classified into pure and complex forms, with additional neurological symptoms like seizures, intellectual disability or movement disorders in complex HSP [3]. Diagnostic yields by NGS range from 28% in simplex to 49% in complex HSP [3, 4].

Whole genome sequencing (WGS) provides the chance to overcome some technical limitations of WES and to identify variants across the entire genome. However, noncoding variant interpretation remains challenging, resulting in only a moderately increased diagnostic yield [5]. Complementary transcriptomic and proteomic analyses thus offer additional functional evidence for variant interpretation [6, 7].

**Figure 1:**
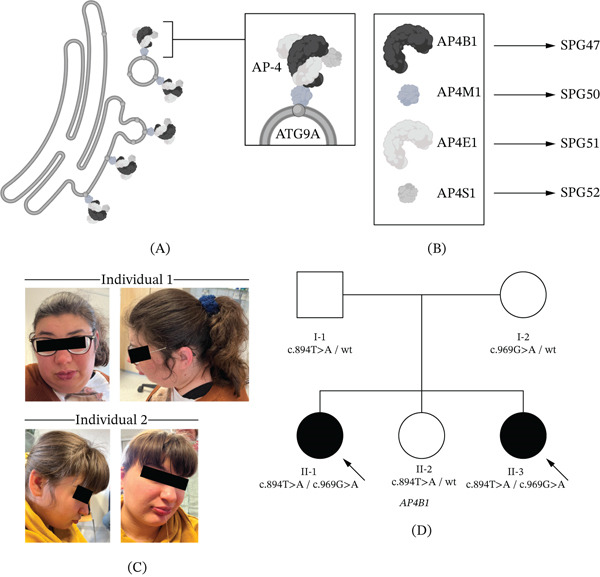
AP‐4‐associated HSP. The heterotetrameric AP‐4‐complex mediates trafficking of cargo proteins, e.g. ATG9A, from the trans‐Golgi network to the cell periphery (A). Biallelic variants in any of the genes encoding for AP‐4 subunits lead to deficiency of the entire AP4‐complex and cause similar manifestations of complex HSP (B). Both affected sisters show nonspecific dysmorphic features, including an everted lower lip, and a dropped head due to muscular hypotonia (C). Pedigree of the family showing an autosomal recessive inheritance pattern (D); parents non‐consanguineous; genotype *AP4B1* (NM_006594.5); healthy individuals have confirmed carrier status.

The heterotetrameric AP‐4‐complex mediates vesicular trafficking of cargo proteins, including ATG9A, from the trans‐Golgi network to neuronal cell periphery (Figure 1A), where ATG9A contributes to autophagosome biogenesis essential for axonal integrity [8]. Biallelic loss‐of‐function variants in AP‐4 subunits cause similar manifestations of an autosomal recessive childhood‐onset complex HSP: SPG47 (*AP4B1*), SPG50 (*AP4M1*), SPG51 (*AP4E1*), and SPG52 *(AP4S1*), summarized as AP‐4‐associated HSP [8](Figure 1B). Affected infants present with muscular hypotonia, postnatal microcephaly, developmental delay, and often seizures. Over time, hypotonia transitions into spasticity. Most individuals show moderate to severe intellectual disability and reduced mobility. Complications in advanced stages of the disease include contractures, foot deformities, dysphagia, bladder, and bowel dysfunction [9].

Using a multiomics workflow we identified the genetic cause in two sisters with complex childhood‐onset spastic paraplegia.

## 2. Methods

### 2.1. Patients and samples

Two affected sisters were assessed in Munich, Germany. Peripheral blood for DNA and RNA extraction and fibroblast cultures from skin biopsies were obtained from the sisters and their parents. Clinical data were reviewed using HPO terminology. Written informed consent including publication of images was provided. WGS, RNA sequencing, and proteomics were performed within the Bavarian Genomes consortium project. All procedures were approved by the local ethics committee of TUM University Hospital (589/20 S) and in accordance with the Declaration of Helsinki.

### 2.2. Whole genome sequencing

DNA was isolated from peripheral blood. For DNA library preparation the Illumina DNA PCR‐Free Library kit was used and sequencing was performed on an Illumina NovaSeq6000 system (Illumina, San Diego, California, United States) to an average of more than 45‐fold coverage as 150 bp paired end run (98% of the target sequences were sequenced at least 20 times). Alignment of the reads was performed to the human reference assembly GRCh37 (hg19). GRCh37 was retained to ensure consistency with our established bioinformatic pipeline and in‐house reference datasets, thereby enabling direct comparison across cohorts and analyses. Variants were analyzed using EVAdb (https://github.com/mri-ihg/EVAdb). Variant classification was determined according to American College of Medical Genetics and Genomics (ACMG) interpretation standards [10].

### 2.3. RNA sequencing

RNA was obtained from PAX blood of the patients and their parents with the RNeasy mini kit (Qiagen). Strand‐specific RNA‐seq library preparation was performed according to the TruSeq Stranded mRNA Sample Prep LS Protocol (Illumina) using 1 *μ*g of RNA. Sequencing was performed as 100 bp paired‐end runs on an Illumina HiSeq6000 platform. Reads from RNA‐seq were mapped with STAR v.2.4.2a to hg19. DROP17 (v1.4.0; [11]) was applied to RNA seq data, incorporating OUTRIDER (v1.20.1; [12]) for aberrant expression and FRASER2 (v1.99.4; [13]) for aberrant splicing detection. A control cohort of 350 PAX blood samples from patients with mendelian disorders from our in‐house database was included. The OUTRIDER output for the AP‐4‐complex subunits is provided in supplementary Table S1.

### 2.4. Proteomics

Cellular proteomics was performed on fibroblasts of the affected sisters and their parents. Fibroblast pellets (0.5 × 106 cells) were lysed in 8 M urea buffer. Protein extracts (15 *μ*g) were reduced (10 mM DTT), alkylated (55 mM chloroacetamide), and digested with Trypsin Gold (Promega) at an enzyme‐to‐protein ratio of 1:50. Peptide digests were acidified, desalted, and labeled with TMT 11‐plex reagents (Thermo Fisher Scientific) according to Zecha et al. [14]. Each batch comprised 10 patient samples and one common reference sample for cross‐batch normalization. Labeled peptides were off‐line fractionated using basic reverse phase chromatography (XBridge Peptide BEH C18, 130 Å, 3.5 *μ*m, 2.1 mm x 150 mm, Waters). Peptides were eluted with increasing acetonitrile concentration into 96 fractions, which were subsequently pooled to 32 fractions. Each fraction was measured with a 60 min linear gradient from 8%‐35% solvent B (0.1% FA, 5% DMSO in acetonitrile) in solvent A (0.1% FA, 5% DMSO in HPLC grade water) on an Ultimate 3000 RSLCnano system coupled to a Fusion Lumos Tribrid mass spectrometer (Thermo Fisher Scientific) in data‐dependent acquisition and multi‐notch MS3 mode [15]. Protein identification was performed using MaxQuant v1.6.17.0. Data analysis was performed with PROTRIDER (https://github.com/gagneurlab/PROTRIDER) as described by Klaproth‐Andrade et al. [16]. The software uses an autoencoder‐based method to detect protein outliers from mass spectrometry‐based intensities (249 fibroblasts from individuals with suspected Mendelian disorders from our in‐house database), providing multiple testing‐corrected p‐values using the Benjamini‐Yekutieli procedure (p_adj_ corresponding to a significance level of 0.05) and fold‐change (fc) for deviations from the expected values. The PROTRIDER output for the AP‐4‐complex subunits and the cargo protein ATG9A is provided in supplementary Table S2.

## 3. Results

### 3.1. Clinical presentation of complex HSP

The two sisters exhibited a complex syndromic disorder characterized by short stature, microcephaly, partial agenesis of the corpus callosum, seizures, progressive spasticity, and cognitive impairment. They are the first (Individual 1; 37 years) and third daughter (Individual 2; 27 years) of healthy, non‐consanguineous parents. The second daughter is healthy.

After an unremarkable pregnancy and birth, both affected daughters showed muscular hypotonia that evolved in a progressive spastic paraplegia, postnatal microcephaly and delayed development. The older sister was sitting at 2 years and crawling at 4 years of age while free walking was never achieved. Moreover, speech and intellectual development was impaired. Both sisters do not speak more than a few words and show severe intellectual disability. The older sister had febrile seizures from 8 months until the 5^th^ year of life. She did not receive anti‐seizure therapy. The younger sister developed a focal status epilepticus at the age of one year during a febrile viral infection. She did not require anti‐seizure medication and seizures were self‐limiting at 13 years. Cerebral MRIs, performed at the age of one year in both sisters, respectively, showed a hypoplasia of the anterior and a partial agenesis of the posterior part of the corpus callosum and delayed myelinization in both. In addition, MRI revealed a bilateral temporo‐frontal cortical and subcortical atrophy and enlarged ventricles and external CFS spaces in individual 2. Further clinical manifestations in both sisters include short stature with a height of 150 cm, nonspecific dysmorphic features (Figure 1C), hypersalivation, and urinary and fecal incontinence. The older sister has myopia and reduced vision, the younger sister shows a nystagmus. Due to contractures, both have undergone several orthopedic surgeries and treatment with intramuscular botulinum toxin. Supportive therapy includes occupational therapy, physiotherapy, and logopedics. An emerging complication is progressive dysphagia.

Shared symptoms and a similar clinical course of disease suggested a monogenic disorder with autosomal recessive inheritance (Figure 1D). No additional cases exist in the family.

### 3.2. Previous genetic diagnostics suggested digenic inheritance

Array‐CGH was unremarkable. Quattro‐WES had identified two monoallelic pathogenic nonsense variants in *AP4S1* (NM_001128126.3:c.124C > T, maternally inherited) and *AP4B1* (NM_006594.5:c.894 T > A, paternally inherited) in both sisters. A synonymous *AP4B1* variant (NM_006594.5:c.969G > A, maternally inherited) was also observed but classified as “likely benign” according to ACMG criteria PM2, BP4, BP7. As both genes encode AP‐4 subunits, a digenic inheritance was hypothesized.

### 3.3. OMICs diagnostics establishes diagnosis of *AP4B1*‐associated HSP

**Figure 2:**
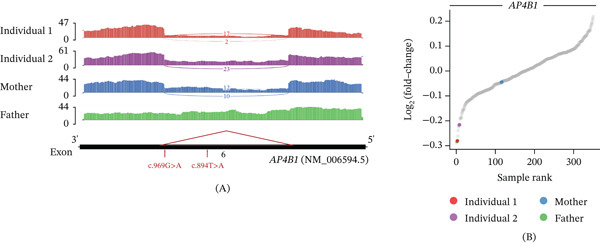
RNA sequencing detected aberrant splicing caused by the synonymous variant and reduced RNA expression due to the nonsense variant in *AP4B1*. Sashimi‐plot visualization of blood RNA‐seq data of the sisters and their parents showed aberrant splicing resulting from the maternally inherited variant c.969G > A leading to a loss of 222 nucleotides (inframe deletion of 74 aminoacids) in exon 6 of *AP4B1* (A). The sample rank plot shows reduced RNA expression of carriers of the nonsense variant c.894 T > A (Ind. 1: p_adj_ = 1, fc = 0.82; Ind. 2: p_adj_ = 1, fc = 0.86; father: p_adj_ = 1, fc = 0.82) compared to 350 other transcriptome datasets (B).

**Figure 3:**
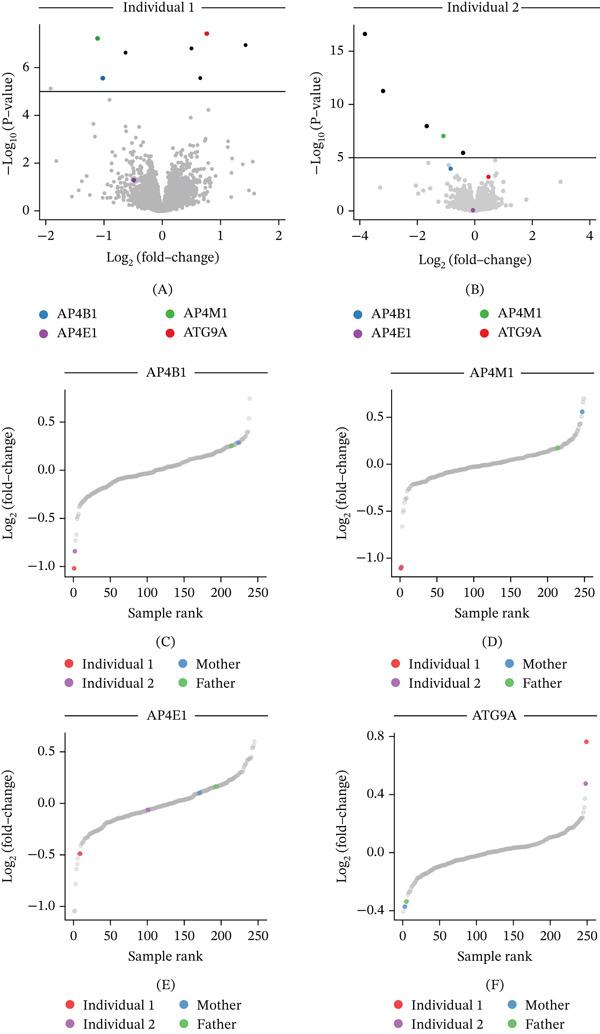
Proteomic analysis detected a reduction of AP‐4‐complex components and an upregulation of the cargo protein ATG9A in the affected sisters. Volcano plots of the proteomic analysis on cultured fibroblasts showed a reduction of AP4B1 and AP4M1 and an upregulation ATG9A in individual 1 (A; AP4B1: p_adj_ = 0.026, fc = 0.49; AP4M1: p_adj_ = 0.002, fc = 0.46; ATG9A: p_adj_ = 0.002, fc = 1.7) and 2 (B; AP4B1: p_adj_ = 0.078, fc = 0.56; AP4M1: p_adj_ = 0.0015, fc = 0.47; ATG9A: p_adj_ = 1, fc = 1.39). AP4E1 was barely reduced (not statistically significant; Ind. 1: p_adj_ = 1, fc = 0.71; Ind. 2: p_adj_ = 1, fc = 0.96) while AP4S1 was not detectable in the proteome data. Other significant outliers are shown in black. Horizontal lines depict the corrected significance level. The sample rank plots show that AP4B1 (C) as well as AP4M1 abundance (D) of the affected sisters were the lowest of 249 in‐house fibroblast proteome datasets. AP4E1 was not identified as an outlier (E), while ATG9A abundance of the sisters was the highest in the cohort (F).

WGS confirmed the previously reported variants. The nonsense variant in *AP4B1* c.894 T > A was classified as “pathogenic” (PVS1, PM2_supporting, PM3, PP4, PP5). SpliceAI predicted the formation of a new splice acceptor site two nucleotides downstream (*Δ* score = 0.99) and a new splice donor site 221 nucleotides upstream (*Δ* score = 0.54) of the synonymous *AP4B1* variant. RNA sequencing confirmed a loss of 222 nucleotides in exon 6, resulting in an in‐frame deletion of 74 amino acids (p.(Gly250_Ser323del)) (Figure 2A). *AP4B1* transcript levels were reduced in carriers of the nonsense variant compared to 350 control blood transcriptomes, even though not statistically significant (Ind. 1: p_adj_ = 1, fc = 0.82; Ind. 2: p_adj_ = 1, fc = 0.86; father: p_adj_ = 1, fc = 0.82; Figure 2B; supplementary Table S1). The *AP4S1* variant resulted in nonsense‐mediated decay of the transcripts as well (Ind. 1: p_adj_ = 1, fc = 0.68; Ind. 2: p_adj_ = 1, fc = 0.67; mother: p_adj_ = 1, fc = 0.63), but no second variant was identified in this gene. Proteomic analysis of the AP‐4‐complex revealed reduced abundance of AP4B1 (statistically significant in individual 1; Ind. 1: p_adj_ = 0.026, fc = 0.49; Ind. 2: p_adj_ = 0.78, fc = 0.56) and AP4M1 (statistically significant in individual 1 and 2; Ind. 1: p_adj_ = 0.002, fc = 0.46; Ind. 2: p_adj_ = 0.0015, fc = 0.47) and a minimal reduction of AP4E1 (not statistically significant; Ind. 1: p_adj_ = 1, fc = 0.71; Ind. 2: p_adj_ = 1, fc = 0.96) in the affected sisters in comparison with 249 controls (Figure 3A,B,C,D,E; supplementary Table S2). AP4S1 was not detected in the affected sisters, their parents and a subset of controls by proteomics. In line with the reduction of AP‐4‐complex components, ATG9A, cargo of the AP‐4‐complex, was upregulated (statistically significant in individual 1; Ind. 1: p_adj_ = 0.002, fc = 1.7; Ind. 2: p_adj_ = 1, fc = 1.39; Figure 3 A,B,F; supplementary Table S2).

Based on these findings from multiomics diagnostics, the synonymous variant was reclassified as “likely pathogenic” (PM2_supporting, PM3, PM4, PP4), and the presence of biallelic (likely) pathogenic variants in *AP4B1* established the diagnosis of monogenic “Spastic paraplegia 47, autosomal recessive” (OMIM #614066), while the hypothesis of digenic inheritance was refuted.

## 4. Discussion

Given the high number of variants that can be detected in each individual by WGS, the *a priori* probability of any single genetic variant to have a causal association with the patient’s clinical phenotype is very low. Thus, variant interpretation can be challenging and result in misclassification, even in the context of suggestive *in silico* prediction scores. This risk can be reduced by integrating multiomics data (e.g. transcriptomics and proteomics) in diagnostic workflows that provide functional evidence of downstream effects of genetic variants [6].

In this study, multiomics including transcriptomic and proteomic analysis established the diagnosis of *AP4B1*‐associated HSP in two sisters with complex spastic paraplegia and refuted the initial hypothesis of digenic inheritance. RNA‐sequencing demonstrated aberrant splicing caused by a synonymous variant in *AP4B1* (Figure 2A), enabling its reclassification from “likely benign” to “likely pathogenic”.

As most synonymous variants are benign, exceptions are often overlooked as possible disease causes. In rare cases, synonymous variants affect splicing, translational efficiency, or mRNA stability, and should be considered in the diagnostic process [17]. *In silico* predictions may indicate possible splice effects of synonymous variants [18], but they are not reliable enough to establish a diagnosis.

RNA sequencing can identify aberrant expression, aberrant splicing and monoallelic expression of rare variants [19]. By providing functional evidence, *in silico* predictions can be validated and variants of uncertain significance or incorrectly as benign considered variants can be reclassified. The complementary implementation of RNA sequencing to WES or WGS has shown to increase the diagnostic rate of mendelian disorders by 7.5‐36% [19–21].

Quantitative proteomics of fibroblasts from patients with suspected monogenic disorders further improves the diagnostic yield in WES or WGS negative cases [6, 22, 23]. Proteomic data detect not only aberrant protein expression due to a pathogenic variant in the corresponding gene, but also functional consequences by alterations of protein complexes and downstream interaction partners [24]. Biallelic variants in each subunit of the AP‐4‐complex lead to a similar HSP phenotype suggesting that all subunits are necessary for its function [8].

Proteomic analysis in this study showed a reduced abundance of AP‐4 subunits AP4B1 and AP4M1 (Figure 3A,B,C,D) along with an upregulation of the AP‐4 cargo protein ATG9A (Figure 3A,B,F). As causative variants were identified only in *AP4B1*, these alterations are considered consequences of AP‐4‐complex deficiency. AP4E1 abundance showed no relevant changes (Figure 3A,B,E). Despite AP4S1 being generally expressed in fibroblasts, it was not detected in the affected sisters and their parents and could be misinterpreted as lacking expression in these individuals. However, comparison with the control cohort shows that 63/249 individuals had missing raw intensities for AP4S1 suggesting a methodological limitation rather than a biological effect.

Consistent with our fibroblast proteomic data, a compensatory upregulation of ATG9A has also been shown by Western blot analyses in patient‐derived fibroblasts and iPSC‐derived neurons from individuals with *AP4B1*‐associated HSP, with ATG9A accumulating in the trans‐Golgi network due to AP‐4 dysfunction [25]. In mouse models, AP‐4 deficiency causes impaired neurite outgrowth, brain malformations including dysgenesis of the corpus callosum [26], and an impairment of autophagy with axonal swelling [27], underlying the neurodevelopmental and neurodegenerative components of the disease. Proteomic analyses thus provide insights into pathophysiology by linking genetic variation to downstream biological effects.

Based on the mislocalization of ATG9A in AP‐4 deficiency, an imaging assay in patient‐derived fibroblasts has been developed that quantifies the subcellular distribution of ATG9A using the “ATG9A ratio”, defined as the fluorescence intensity of ATG9A within the trans‐Golgi network relative to the remaining cell body [28]. This measure serves as a surrogate marker of AP‐4 complex dysfunction and has recently shown to support the interpretation of variants of uncertain significance in AP‐4 subunit genes [29]. However, because an elevated ATG9A ratio reflects dysfunction of the entire AP‐4 complex, the assay does not identify the affected AP‐4 subunit. This represents a limitation in complex genetic cases, such as the family described in this study, in which multiple variants in AP‐4 subunits were prioritized. In this context the use of complementary transcriptomic and proteomic data was necessary to determine the precise molecular cause.

An accurate subtype‐specific molecular diagnosis is becoming increasingly important as gene‐specific AAV‐mediated replacement therapies for AP‐4‐associated HSP are entering clinical evaluation. Recently, an AAV‐mediated gene replacement therapy was successfully applied in a child with *AP4M1*‐associated HSP (SPG50), demonstrating a favorable safety profile and stabilization of disease progression within 12 months after administration [30]. Clinical trials of AAV9‐based gene therapies for SPG50 are currently ongoing (ClinicalTrials.gov IDs NCT05518188 and NCT06692712). In parallel, an AAV9‐mediated gene replacement therapy for *AP4B1*‐related HSP (SPG47) has advanced to clinical evaluation (phase 1/2 clinical trial, ClinicalTrials.gov ID NCT06948019) following promising preclinical efficacy and safety studies that showed a restoration of key hallmarks of disease, including ATG9A mislocalization and motor dysfunction [8]. To ensure affected families access to clinical trials establishing a timely and subtype‐specific diagnosis is critical as enrollment requires molecular confirmation of biallelic pathogenic variants in the specific AP‐4 subunit and focuses on young children, where therapeutic effects are expected to be greatest.

## 5. Conclusion

A multiomics approach enabled the diagnosis of *AP4B1*‐associated HSP in two sisters after years of uncertainty. Early diagnosis is crucial for patients and their families for emotional and practical reasons, facilitating family planning and appropriate clinical management, particularly as AAV‐based gene replacement therapies for AP‐4‐related HSP are currently under clinical evaluation. While RNA sequencing is increasingly integrated into diagnostics, proteomics remains largely confined to research settings [23]. This study demonstrates the value of integrating both methods into the diagnostic evaluation to resolve NGS negative cases and improve patient care.

## Author Contributions

Susann Badmann, Alice Saparov, Michael Zech, Matias Wagner: Study design and data interpretation. Susann Badmann, Alice Saparov, Philip Harrer: Data visualization. Juliane Beuschlein in, Cornelia Daumer‐Haas: Phenotypic characterization. Elisabeth Graf, Christina Ludwig, Julia Mergner: Data acquisition. Thomas Meitinger, Juliane Winkelmann: Funding acquisition and supervision. Susann Badmann: Writing – original draft. Alice Saparov, Theresa Brunet, Maureen Jacob, Holger Prokisch, Michael Zech, Matias Wagner: Writing – Review and Editing. All authors have read and approved the final manuscript. Michael Zech, and Matias Wagner contributed equally to this work.

## Funding

Bavarian Genomes Network for Rare Disorders; Else Kröner‐Fresenius‐Stiftung, 10.13039/501100003042, 2022_EKSE.185; EJP RD (EJP RD Joint Transnational Call 2022) and the German Federal Ministry of Education and Research (BMBF, Bonn, Germany), PREdictive biomarkers in DYsTonia, 01GM2302; Free State of Bavaria under the Excellence Strategy of the Federal Government and the Länder; Technical University of Munich ‐ Institute for Advanced Study, Open Access funding enabled and organized by Projekt DEAL.

## Conflicts of Interest

None of the authors have a conflict of interest to disclose.

## Supporting information


**Supporting Information** Additional supporting information can be found online in the Supporting Information section. OUTRIDER results of blood transcriptomic data for AP‐4‐complex subunits are provided in supplementary Table S1. PROTRIDER results of fibroblast proteomic data for AP‐4‐complex subunits and the cargo protein ATG9A are provided in supplementary Table S2.

## Data Availability

The data that support the findings of this study are available on request from the corresponding author. The data are not publicly available due to privacy or ethical restrictions.
