# Supplementary material for: Splice effect of a synonymous variant in AP4B1: multiomics approach establishes the diagnosis in two sisters with spastic paraplegia
Source: Hum Mutat. 2026 Aug 12;2026:8645235. doi: 10.1155/humu/8645235 (PMC13468907; doi:10.1155/humu/8645235)
Supplement: Supplementary file 1 [file HUMU-2026-8645235-s001.docx]

**Supplement to “Splice effect of a synonymous variant in *AP4B1*: multiomics approach establishes the diagnosis in two sisters with spastic paraplegia”**

**Table S1 OUTRIDER results of blood transcriptomic data for AP-4-complex subunits.**

| Sample | Gene | Raw counts | log2fc | fc | nominal p-value | p_adj_ |
| --- | --- | --- | --- | --- | --- | --- |
| Individual 1 | *AP4B1* | 360 | -0.28 | 0.82 | 0.005 | 1 |
|  | *AP4E1* | 121 | -0.11 | 0.93 | 0.529 | 1 |
|  | *AP4M1* | 140 | -0.03 | 0.98 | 0.915 | 1 |
|  | *AP4S1* | 88 | -0.56 | 0.68 | 8.05E-04 | 1 |
| Individual 2 | *AP4B1* | 485 | -0.22 | 0.86 | 0.019 | 1 |
|  | *AP4E1* | 225 | -0.03 | 0.98 | 0.877 | 1 |
|  | *AP4M1* | 165 | 0.03 | 1.02 | 0.864 | 1 |
|  | *AP4S1* | 122 | -0.58 | 0.67 | 7.31E-05 | 1 |
| Mother | *AP4B1* | 391 | -0.04 | 0.97 | 0.669 | 1 |
|  | *AP4E1* | 84 | -0.29 | 0.82 | 0.139 | 1 |
|  | *AP4M1* | 128 | -0.12 | 0.92 | 0.508 | 1 |
|  | *AP4S1* | 63 | -0.66 | 0.63 | 4.54E-04 | 1 |
| Father | *AP4B1* | 383 | -0.28 | 0.82 | 0.004 | 1 |
|  | *AP4E1* | 136 | -0.05 | 0.97 | 0.783 | 1 |
|  | *AP4M1* | 173 | 0.2 | 1.15 | 0.172 | 1 |
|  | *AP4S1* | 119 | -0.11 | 0.93 | 0.488 | 1 |

**Table S2 PROTRIDER results of fibroblast proteomic data for AP-4-complex subunits and the cargo protein ATG9A.**

| Sample | Protein | Raw intensity | log2fc | fc | nominal p-value | p_adj_ | protein outlier |
| --- | --- | --- | --- | --- | --- | --- | --- |
| Individual 1 | AP4B1 | 3916 | -1.02 | 0.49 | 2.82E-06 | 0.026 | TRUE |
|  | AP4E1 | 52616 | -0.49 | 0.71 | 0.050 | 1 | FALSE |
|  | AP4M1 | 9033 | -1.11 | 0.46 | 6.18E-08 | 0.002 | TRUE |
|  | AP4S1 | NA | NA | NA | NA | NA | FALSE |
|  | ATG9A | 713270 | 0.76 | 1.70 | 3.89E-08 | 0.002 | TRUE |
| Individual 2 | AP4B1 | 3814 | -0.84 | 0.56 | 1.09E-04 | 0.775 | FALSE |
|  | AP4E1 | 56215 | -0.06 | 0.96 | 0.849 | 1 | FALSE |
|  | AP4M1 | 7622 | -1.09 | 0.47 | 9.15E-08 | 0.001 | TRUE |
|  | AP4S1 | NA | NA | NA | NA | NA | FALSE |
|  | ATG9A | 531170 | 0.48 | 1.39 | 6.34E-04 | 1 | FALSE |
| Mother | AP4B1 | 10675 | 0.29 | 1.22 | 0.199 | 1 | FALSE |
|  | AP4E1 | 66849 | 0.10 | 1.07 | 0.622 | 1 | FALSE |
|  | AP4M1 | 28111 | 0.56 | 1.47 | 0.007 | 1 | FALSE |
|  | AP4S1 | NA | NA | NA | NA | NA | FALSE |
|  | ATG9A | 229930 | -0.37 | 0.77 | 0.007 | 1 | FALSE |
| Father | AP4B1 | 8770 | 0.25 | 1.19 | 0.259 | 1 | FALSE |
|  | AP4E1 | 52628 | 0.16 | 1.12 | 0.454 | 1 | FALSE |
|  | AP4M1 | 18316 | 0.18 | 1.13 | 0.402 | 1 | FALSE |
|  | AP4S1 | NA | NA | NA | NA | NA | FALSE |
|  | ATG9A | 261910 | -0.34 | 0.79 | 0.015 | 1 | FALSE |
